# Correlation of Global and Gene-Specific DNA Methylation in Maternal-Infant Pairs

**DOI:** 10.1371/journal.pone.0013730

**Published:** 2010-10-29

**Authors:** Molly L. Kile, Andrea Baccarelli, Letizia Tarantini, Elaine Hoffman, Robert O. Wright, David C. Christiani

**Affiliations:** 1 Environmental Health Department, Harvard School of Public Health, Boston, Massachusetts, United States of America; 2 Department of Environmental and Occupational Health, Center of Molecular and Genetic Epidemiology, Ca' Granda Ospedale Maggiore Policlinico IRCCS Foundation, University of Milan, Milan, Italy; Bellvitge Biomedical Research Institute (IDIBELL), Spain

## Abstract

The inheritance of DNA methylation patterns is a popular theory to explain the influence of parental genetic and environmental factors on the phenotype of their offspring but few studies have examined this relationship in humans. Using 120 paired maternal-umbilical cord blood samples randomly selected from a prospective birth cohort in Bangladesh, we quantified DNA methylation by pyrosequencing seven CpG positions in the promoter region of *p16*, four CpG positions in the promoter region of *p53*, *LINE-1* and *Alu*. Positive correlations were observed between maternal and umbilical cord blood at *p16, LINE-1*, and *Alu* but not *p53*. Multiple linear regression models observed a significant association between maternal and umbilical cord blood at *LINE-1* and *Alu* (*LINE-1*: β = 0.63, p<0.0001; *Alu*: β = 0.28, p = 0.009). After adjusting for multiple comparisons, maternal methylation of *p16* at position 4 significantly predicted methylation at the same position in umbilical cord blood (β = 0.43, p = <0.0001). These models explained 48%, 5% and 16% of the observed variability in umbilical cord %5mC for *LINE-1, Alu* and *p16* at position 4, respectively. These results suggest that DNA methylation in maternal blood was correlated with her offspring at *LINE-1, Alu*, and *p16* but not *p53*. Additional studies are needed to confirm whether these observed associations were due to the inheritance of epigenetic events or the shared environment between mother and fetus. Future studies should also use a multi-generational family-based design that would quantify both maternal and paternal contributions to DNA methylation in offspring across more than one generation.

## Introduction

DNA methylation is an epigenetic modification that involves the covalent addition of a methyl group to a cytosine at the 5′-position of a CpG dinucleotide [1]. CpG dinucleotides are clustered in the promoter regions of genes [2] and in highly repeated elements such as long interspersed nucleotide elements *(LINE-1)* and *Alu* [3], [4]. There are approximately 1.4 million *Alu* repeated elements and a half million *LINE-1* repeated elements in the human genome. The CpG dinucleotides in these repeated elements are typically heavily methylated in order to silence their expression. They are also transposable, that is, expression can lead to insertion into other genomic regions which can result in gene silencing [5], [6]. These interspersed repetitive elements may serve as surrogate markers for global DNA methylation [7]. CpG rich regions are also found in approximately half of the gene promoter regions. Typical CpG islands are not methylated which allows for normal gene transcription [8]. In many complex diseases including cancer, atherosclerosis, Alzheimer's disease, and psychiatric disorders it is common to observe global DNA hypomethylation, as well as, gene specific hypermethylation [9], [10]. Global DNA hypomethylation is associated with genomic instability and gene specific hypermethylation is associated with gene silencing [11], [12].

In early embryogenesis, there are two waves of demethylation which are completed by the morula stage[13]–[15]. These erasures are quickly followed by an increase in *de novo* methylation which allows for the acquisition of imprinted genes and epigenetic programming associated with tissue differentiation [13]–[15]. It is believed that this re-programming of epigenetic marks during embryogenesis ensures that gametes acquire the appropriate sex-specific epigenetic states and that epimutations acquired by the adult germ cells are removed [16].

The heritability of epigenetic marks between generations is frequently used to explain the etiology of traits and diseases that do not follow Mendelian inheritance patterns. Transgenerational inheritance of DNA methylation has been described in plants, yeast, *Drosophila*, and mouse models for both transgenes and endogenous alleles [17]–[20]. However, the inheritance of DNA methylation in humans has only been evaluated in families with a history of cancer. Studies of hereditary nonpolyposis colorectal cancer have observed hypermethylation of DNA mismatch repair genes (*MSH2* and *MLH1*) in the proband and their affected children [21]–[24]. In families with a history of testicular cancer, researchers have observed strong gender-specific *LINE-1* methylation patterns between parents and offspring, particularly between affected father-affected son pairs [25].

To better understand the relationship of epigenetic patterns in parent-offspring pairs, we evaluated DNA methylation patterns in 120 paired maternal-child samples collected in a prospective reproductive health study recruited in Bangladesh. This observational study used pyrosequencing to quantify DNA methylation in peripheral leukocytes at two tumor suppressor genes (*p16* and *p53*) and two repetitive elements (*LINE-1* and *Alu*). The tumor suppressor genes were selected because both *p16* and *p53* have well characterized CpG positions in their promoter regions. Furthermore, *p16* expression is well known to be regulated via DNA methylation [26].

## Results

Average blood DNA methylation levels, expressed at %5mC (percentage of cytosines that are methylated over unmethylated cytosines at a given CpG position), are presented in Table 1. On average, DNA methylation for *LINE-1* was 80.1 (SD = 2.1) and 80.6 (SD = 1.9) and *Alu* was 25.2 (SD = 0.7) and 25.0 (SD = 0.8) in maternal and umbilical cord samples, respectively. Paired t-tests detected very slight differences in %5mC between maternal and umbilical cord blood with umbilical cord blood containing, on average, 0.5% (p = 0.007) more methylated cytosines at *LINE-1* compared to maternal blood. Whereas, maternal blood contained on average 0.25% (p = 0.006) more methylated cytosines at *Alu* compared to umbilical cord blood. Gender specific paired t-tests observed a slight difference at *LINE-1* and *Alu* between mother-daughter pairs but not between mother-son pairs. On average, daughters had 0.4% less DNA methylation at *LINE-1* (M = −0.43, SD = 1.48, p = 0.04), and 0.3% more DNA methylation at *Alu* (M = 0.32, SD = 0.76, p = 0.003) compared to their mothers.

**Table 1:** General descriptive statistics for paired maternal-cord blood samples included in the analysis.

		Maternal Blood		Cord Blood		T-test	
	*n*	*Mean*	*SD*	*Mean*	*SD*	*Dif.*	*p-value*
*Alu*	*103*	25.2	0.71	24.96	0.78	0.25	0.007
*LINE-1*	*98*	80.11	2.10	80.58	1.92	−0.46	0.006
*p16*							
pos1	*100*	2.61	1.65	2.41	1.43	0.20	0.31
pos2	*100*	3.03	1.66	2.83	1.29	0.20	0.25
pos3	*100*	1.35	0.66	1.38	0.76	−0.03	0.75
pos4	*100*	2.18	1.02	2.05	0.98	0.13	0.24
pos5	*100*	2.16	0.70	2.07	0.91	0.09	0.46
pos6	*100*	1.23	0.70	1.30	0.78	−0.07	0.41
pos7	*100*	2.90	2.17	2.38	1.13	0.52	0.02
*p53*							
pos1	*87*	2.80	1.76	2.55	0.90	0.26	0.23
pos2	*87*	7.92	2.52	7.39	2.29	0.54	0.13
pos3	*87*	2.77	0.97	2.41	0.71	0.36	0.008
pos4	*87*	3.83	1.65	3.68	1.28	0.15	0.48

DNA methylation was also measured at 7 and 4 CpG dinucleotides within the promoter regions of *p16* and *p53*, respectively. DNA methylation was very low at all CpG dinucleotides in both *p16* and *p53* (Table 1). This was expected because the promoter regions of these genes have low levels of methylation in healthy individuals. Paired t-tests detected very slight differences in %5mC at position 7 in *p16* with umbilical cord blood containing, on average, 0.5% (p = 0.02) more methylated cytosines at this CpG dinucleotide compared to maternal blood. Gender specific paired t-tests only observed a difference in DNA methylation at position 7 in *p16* in maternal-daughter pairs with daughters having 0.9% more methylation compared to their mothers (M = 0.9, SD = 2.5, p = 0.02). No difference in DNA methylation at any of the 7 CpG dinucleotides in *p16* was observed in mother-son pairs.

Paired t-tests detected a very slight difference in %5mC at position 3 in *p53* with umbilical cord blood containing, on average, 0.2% (p = 0.008) more methylated cytosines compared to maternal blood. Gender specific paired t-tests observed a difference in DNA methylation at position 2 and position 3 in *p53* in maternal-daughter pairs with daughters having 0.8% and 0.5% more methylation at position 2 and 3 compared to their mother (*p53* position 2: M = 0.77; SD = 2.61, p = 0.05; *p53* position 3 M = 0.48; SD = 1.28, p = 0.01). No differences in DNA methylation at any of the 4 CpG dinucleotides in *p53* was observed in maternal-son pairs. These results suggested that there were gender-specific differences in DNA methylation in *p53* and daughters had slightly less DNA methylation at *LINE-1* and slightly more DNA methylation at *Alu, p16* and *p53* compared to their mothers.

Significant correlations were observed between DNA methylation in maternal-umbilical cord pairs (Table 2). Positive correlations were observed between maternal-umbilical cord pairs at *LINE-1* (σ_s_ = 0.63, p<0.0001), *Alu* (σ_s_ = 0.31, p<0.0001), in *p16* (*p16* position 1: σ_s_ = 0.38, p<0.0001; *p16* position 2: σ_s_ = 0.49, p<0.0001; *p16* position 3: σ_s_ = 0.35, p = 0.0004; *p16* position 4: σ_s_ = 0.54, p<0.0001; *p16* position 5: σ_s_ = 0.17, p = 0.09; *p16* position 6: σ_s_ = 0.46, p<0.0001; *p16* position 7: σ_s_ = 0.41, p<0.0001;). A positive correlation was observed at position 4 in *p53* but not at any of the other 3 positions tested (*p53* position 1: σ_s_ = 0.13, p = 0.24; *p53* position 2: σ_s_ = 0.13, p = 0.22; *p53* position 3: σ_s_ = −0.07, p = 0.54; *p53* position 4: σ_s_ = 0.22, p = 0.04). It is interesting to note that *LINE-1* was positively correlated with *p16* and *p53*, but negatively correlated with *Alu* despite the fact that they are both used as surrogate markers of global methylation status. To test whether the observed regression results would be similar in unrelated individuals, the samples were randomly re-assigned so that the paired samples were no longer related. In the randomly re-assigned data, there was no correlation between maternal-umbilical cord samples at *LINE-1*, *Alu*, *p16* or *p53* (data not shown).

**Table 2:** Spearman correlation coefficients between umbilical cord blood and maternal blood for each epigenetic marker.

			Umbilical Cord Blood												
			*Line-1*	*Alu*	*p16*							*p53*			
					Pos 1	Pos 2	Pos3	Pos4	Pos 5	Pos 6	Pos 7	Pos 1	Pos 2	Pos 3	Pos 4
**Maternal Blood**	*Line-1*		0.63†	−0.25‡	0.24‡	0.49†	0.44†	0.42†	0.11	0.39†	0.44†	0.20‡	−0.03	0.14	0.33†
	*Alu*		−0.26†	0.31†	−0.04	−0.16	−0.1	−0.13	−0.03	−0.17	−0.27†	−0.08	0.1	0.01	−0.28†
	*p16*	Pos 1	0.28†	−0.03	0.38†	0.36†	0.41†	0.42†	0.20‡	0.41†	0.41†	0.11	−0.11	−0.05	0.13
		Pos 2	0.44†	−0.21‡	0.44†	0.49†	0.45†	0.51†	0.16	0.42†	0.47†	0.17	−0.16	−0.09	0.27†
		Pos 3	0.42†	−0.11	0.35†	0.39†	0.35†	0.41†	0.26†	0.41†	0.42†	0.14	−0.18	−0.09	0.2
		Pos 4	0.31†	0.03	0.46†	0.42†	0.47†	0.54†	0.16	0.41†	0.43†	0.17	−0.06	0	0.20‡
		Pos 5	0.09	0.14	0.33†	0.23‡	0.32†	0.30†	0.17	0.28†	0.19‡	0.02	−0.01	0.03	−0
		Pos 6	0.47†	−0.13	0.37†	0.46†	0.42†	0.46†	0.22‡	0.46†	0.43†	0.13	−0.14	−0.08	0.19
		Pos 7	0.35†	−0	0.37†	0.43†	0.42†	0. 46†	0.18	0.41†	0.41†	0.13	−0.13	−0.13	0.15
	*p53*	Pos 1	0.05	−0.1	−0.03	−0.07	−0.1	−0.02	−0.1	−0.1	−0.03	0.13	0.13	0.05	−0.12
		Pos 2	−0.04	0.06	−0.05	−0.13	−0.1	−0.16	−0.04	−0.07	−0.14	0.05	0.13	0.04	−0.09
		Pos 3	0.12	−0.18	0.34†	0.21‡	0.25‡	0.28†	0.14	0.24‡	0.24‡	0.1	−0.02	−0.07	−0.02
		Pos 4	0.07	−0.07	0.26‡	0.18	0.23‡	0.28†	0.05	0.23‡	0.22‡	0.15	−0.04	0.03	0.22‡

‡ 0.05≥p≥0.01.

† 0.01>p>0.0001.

**Figure 1:**
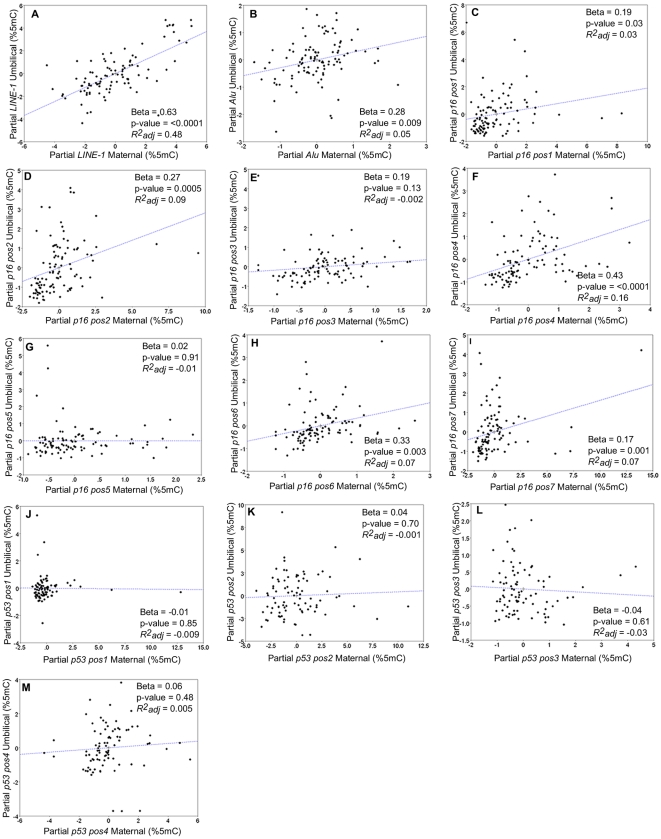
Partial regression plots including the effect estimate and p-value from multiple regression analysis that test the association between %5mC in umbilical cord and maternal blood at LINE-1. (Panel A), Alu (Panel B), seven CpG positions in the p16 promoter (Panels C–I), and four CpG positions in the p53 promoter (Panels J–M).

Multiple linear regression models evaluated whether the %5mC in maternal blood significantly predicted %5mC in umbilical cord blood (Figure 1 A–M). These models adjusted for infant sex, mother's age, and arsenic exposure in the mother's drinking water during pregnancy. Maternal methylation of *LINE-1* and *Alu* significantly predicted umbilical cord %5mC in *LINE-1* and *Alu*, respectively (Figure 1A and 1B: β = 0.63, p<0.0001; β = 0.28, p = 0.009). These models explained 48% and 5% of the observed variability in umbilical cord DNA methylation at *LINE-1* and *Alu*. At 6 of the 7 CpG positions screened in *p16*, the %5mC in maternal blood significantly predicted the %5mC in the corresponding CpG positions in the umbilical cord blood (Figure 1C *p16* position 1: β = 0.19, p = 0.003; Figure 1D *p16* position 2: β = 0.27, p = 0.0005; Figure 1F *p16* position 4: β = 0.43, p<0.0001; Figure 1H *p16* position 6: β = 0.33, p = 0.003; Figure 1I *p16* position 7: β = 0.17, p = 0.001). These models explained 3%, 9%, 16%, 7%, and 7% of the observed variability in umbilical cord DNA methylation at *p16* position 1, 2, 4, 6, and 7, respectively. Using a more stringent α = 0.007 to account for the potential false positives resulting from multiple comparisons of CpG positions within the promoter region of *p16*, only the %5mC at position 4 in maternal blood remained highly significant. Maternal methylation of *p53* was not a significant predictor of umbilical cord %5mC at any of the 4 CpG dinucleotides assayed (Figures 1J–M).

## Discussion

Unlike DNA sequence mutations, the inheritance patterns of epigenetic events in humans are poorly understood. This epidemiological study observed that DNA methylation levels in *LINE-1, Alu*, and *p16* appeared to be positively associated in healthy mother-infant pairs. However, evaluating changes in epigenetic patterns from one generation to the next must be interpreted cautiously because such marks are both cell specific and malleable. Many factors have been shown to influence DNA methylation including gender [27], aging [28], [29], environmental factors [30], [31], and heterogeneous peripheral blood leukocyte populations [32], [33]. In addition, the timing of the measurement, cell type, external environment and the function of the mark (i.e. gene expression regulation which changes with life stage) could influence the observed pattern. Aside from imprinted genes, the evidence that some epigenetic marks are inherited across generations comes largely from animal models [34]. For example, in mice the transgenerational epigenetic inheritance of the *agouti viable yellow* (*A ^vy^*) allele and the *axin-fused* (*Axin ^Fu^*) allele, which both include a IAP retrotransposon in their sequence, has been demonstrated [35], [36].

Few studies have investigated transgenerational patterns of epigenetic marks in humans and these have mostly been limited to families with a history of disease. For instance, in families with a history of hereditary nonpolyposis colorectal cancer, there is evidence of heritable germline inheritance of hypermethylated promoter region in DNA mismatch repair genes including *mutL homolog 1* (*MLH1*) and *mutL homolog 2* (*MLH2*) alleles that suggests individuals who inherited these epimutations have a predisposition to this particular type of cancer [22], [23], [37]. Another study in families with a history of testicular cancer reported that global methylation at *LINE-1* in peripheral blood of offspring were significantly positively correlated with parental levels, particularly between mother-daughter (*r* = 0.48, p-value = <0.001), father-daughter (*r* = 0.31, p-value = 0.02), and affected father-affected son pairs (*r* = 0.49, p-value = 0.03) [25]. Two additional studies also suggest that global methylation patterns may be inherited. Hillemacher *et al*, who compared DNA methylation in 73 fathers, 69 mothers and 156 grown offspring, reported an association between offspring's and paternal DNA methylation if both had never smoked *(r* = 0.41, β = 0.68, *p* = 0.02) [38]. Sandovici *et al* conducted a study of three-generation families and reported familial clustering of high methylation at *Alu* amongst individuals who came from families in which one member exhibited abnormal patterns of methylated regions of the *IGF2/H19* or *IGF2R* loci [39].

In this study, the strongest association in maternal-infant pairs was with *LINE-1*. Furthermore, the strength of the correlation observed (*r* = 0.48) was similar that observed in the families with a history of testicular cancer [25]. Although it is interesting to note that human *LINE-1* elements include an intracisternal A particle (IAP) retrotransposon in their sequence that is very similar to the IAP which determines epigenetic inheritance in the A^vy^ and Axin^fu^ animal models [40]. This could explain the strong parent-offspring associations observed by both Mirabello et al [25] and this study. However, it should be noted that the association at *LINE-1* reflects an average methylation across over 500,000 loci across the genome and is not specific to correlations between any given loci. Therefore, it is possible that the associations observed in this study reflect a more global methylation capacity which could be due to inherited methyltransferase genes.

We also observed that *LINE-1* and *Alu* methylation levels were inversely associated with each other. However, studies that have used DNA from tumor samples have shown that the *LINE-1* and *Alu* methylation were correlated with each other [41], [42]. No significant correlations have been reported, to the best of our knowledge, between *LINE-1* and *Alu* methylation levels in non-malignant tissue samples such as blood leukocytes [43], [44]. The finding of a negative correlation between *LINE-1* and *Alu* conflicts with the hypothesis of a direct role of general methyltransferase activities in determining the observed mother-child correlations, and suggest more complex, position-specific mechanisms. There is growing evidence that *Alu* and *LINE-1* have distinct functional roles that may account for different and even inverse methylation patterns within the same subjects as was observed in this study [45]. For instance, there is recent evidence showing that *Alu* and *LINE-1* undergo opposite DNA methylation changes as individuals age [31], [46]. Therefore, our results provide further indirect evidence that *LINE-1* and *Alu* may respond differently or have distinct functional roles in non-malignant tissues.

While it is possible that the observed correlation between maternal-infant DNA methylation patterns is a result of maternal contamination of umbilical cord blood due to leakage between maternal-fetal circulation during pregnancy and/or partition [47], this explanation is unlikely because we did not observe any association between maternal and umbilical cord blood DNA methylation with *p53*. While our multiple linear regression models adjusted for maternal age and the sex of the infant, it is also possible that shared environmental factors between the mother and fetus explain the observed DNA methylation patterns. This population was recruited in Bangladesh as part of a reproductive health study examining the role of arsenic exposure on reproductive health outcomes. While our analysis controlled for arsenic concentration in the mother's drinking water during pregnancy, the effects of arsenic exposure in mothers and fetus cannot be teased apart for obvious reasons. Arsenic is a suspected epigenetic toxicant [48], [49]. Nor did we control for dietary factors that can influence DNA methylation such as folate and homocysteine [50], [51], but again any exposure to a mother will by default occur in the fetus. Also, gene expression is regulated at least in part by DNA methylation. If particular genes (or retrotransposons) need to be expressed in order to preserve cell function at specific life stages, this constitutional need will tend to increase the correlation between subjects.

Another limitation of this study is that we were unable to adjust for the distribution of peripheral blood leukocyte populations in our whole blood samples or the timing of the blood sample collection from the umbilical cord. Also, paternal DNA from blood leucocytes was not collect which prevented us from examining the correlation between paternal DNA methylation and their offspring. Ideally, a case-parent trio design would be employed to examine the degree of DNA methylation between both parents and their offspring.

In conclusion, the results of this study suggest that *LINE-1, Alu* and *p16* DNA methylation in maternal blood collected during pregnancy predicts the DNA methylation patterns in the cord blood of her newborn. We did not find correlation for *p53* methylation. Overall our results are consistent with the hypothesis that some, but not all, DNA methylation marks may be heritable; however, it is also possible that these associations are due to the shared environment unique to the mother and fetus or to constitutional methylation patterns that are necessary for cell function. Multi-generational family-based studies are needed to determine the extent to which *LINE-1, Alu* and *p16* are heritable.

## Materials and Methods

### Subject Selection and Recruitment

This study was approved by the Human Research Committees at the Harvard School of Public Health and Dhaka Community Hospital (DCH). All volunteers provided written consent before participating in the study.

We used 120 paired maternal-umbilical cord blood samples collected as part of an ongoing prospective birth cohort that is investigating the effects of prenatal arsenic exposure on reproductive health outcomes. This study is recruiting pregnant women residing in the Sirajdikhan and Pabna Upazilas of Bangladesh through active surveillance in the districts. Women were eligible for the study if they were 18 years of age or older, had an ultrasound-confirmed singleton pregnancy of less than 28 weeks' gestation, used a tubewell as their primary drinking water source when they conceived, planned to live at their current residence for the duration of the pregnancy, planned to continue prenatal health care with Sirajdikhan Community Clinic a rural health care clinic operated by DCH, and agreed to deliver at DCH or at home with a DCH-trained midwife. All participants were provided with a free supply of prenatal vitamins that was refilled monthly when field staff visited each participant in their home. Informed consent was obtained from all participants before enrollment.

### Exposure Assessment

Water samples were collected from each participant's tubewell at the time of enrollment. Tubewells were purged by pumping the well for several minutes before 50 mls of water was collected in an acid-washed polypropylene tube (BD Falcon, BD Bioscience, Bedford, MA). Samples were preserved with Reagent Grade HNO_3_ (Merck, Germany) to a pH<2 and kept at room temperature until analysis. Arsenic concentrations were quantified by inductively coupled plasma-mass spectrometry using US EPA method 200.8 (Environmental Laboratory Services, North Syracuse, New York). Analysis was validated using PlasmaCAL multi-element QC standard #1 solution (SCP Science, Canada). The average percent recovery for InAs was 102±7%. The limit of detection (LOD) for this method is 1 μg As/L. Samples below the LOD were assigned a value of 0.5 μg As/L.

### Peripheral Blood Collection and DNA extraction

A peripheral whole blood sample was collected from the participant when they enrolled in the study and umbilical cord blood was collected at the time of delivery. DNA was extracted from 4 mls of whole blood using Puregene DNA isolation kits (Qiagen/Gentra Systems, Minneapolis, MN) following manufacturers instructions. Extracted DNA was stored at −20°C until further analysis.

### DNA Methylation

DNA methylation analyses were performed in duplicate on bisulfite-treated DNA using highly-quantitative analysis based on PCR-Pyrosequencing where 0.5 µg DNA (concentration 25 ng/µl) was treated using the EZ-96 DNA Methylation-Gold™ Kit (Zymo Research, Orange, CA, USA) according to the manufacturer's protocol. Final elution was performed with 30 µl M-Elution Buffer.

**Figure 2:**
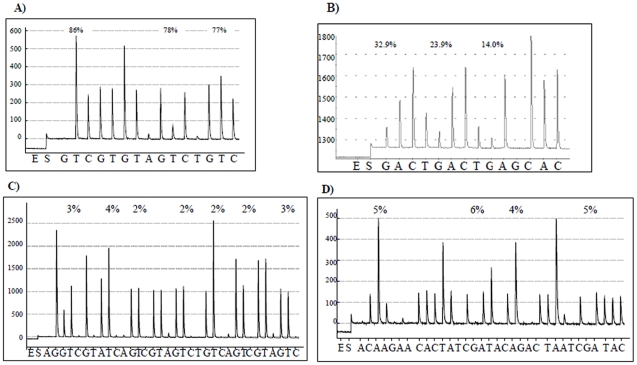
Examples of pyrograms. A) *LINE-1*, B) *Alu*, C) *p16*, and D) *p53*.

In brief, DNA was amplified using bisulfite-PCR where a biotin-labeled primer was used to purify the final PCR product by Streptavidin Sepharose (Amersham Biosciences, Uppsala, Sweden) and the Pyrosequencing Vacuum Prep Tool (Pyrosequencing, Inc., Westborough, MA) as per the manufacturer's recommendations. Then the PCR product underwent pyrosequencing using the PyroMark™Q96 MD Pyrosequencing System (Pyrosequencing, Inc., Westborough, MA) as previously described [52] using 0.3 µΜ sequencing primer. Examples of the pyrograms for each sequence are presented in Figure 2.

The degree of methylation was expressed for each DNA locus as the percentage methylated cytosine over the sum of methylated and unmethylated cytosine. Non-CpG cytosine residues were used as built-in controls to verify bisulfite conversion. Each marker was tested in two replicates and their average was used in the statistical analysis.

To estimate global DNA methylation content we performed DNA methylation analyses of *Alu* and *LINE-1* repeated sequences, which allow for the amplification of a representative pool of repetitive elements, as previously described [30]. *p16* DNA methylation was measured using primers and conditions developed by Shaw et al [53]. We developed the assay for *p53* methylation by locating the *p53* promoter, using the Genomatix Software (Genomatix Software Inc, Ann Arbor, MI). Table 3 shows the localization of gene promoters, regions amplified and CpGs analysed for *p16* and *p53*.

**Table 3:** Localization of gene promoters and regions amplified and of the CpG dinucleotide positions at which DNA methylation was quantified.

Gene	Chromosome	Promoter		Amplicon		CpGs
		*Start*	*End*	*Start*	*End*	
*p16*	9	21964701	21965538	21965321	21965395	21965350 (position 1)
						21965355 (position 2)
						21965357 (position 3)
						21965361 (position 4)
						21965365 (position 5)
						21965368 (position 6)
						21965374 (position 7)
*p53*	17	7531143	7531743	7531409	7531628	7531486 (position 1)
						7531473 (position 2)
						7531469 (position 3)
						7531458 (position 4)

A 50 µL PCR was carried out in 25 µL GoTaq Green Master mix (Promega, Madison, WI, USA), 10 pmol forward primer, 10 pmol reverse primer, 50 ng bisulfite-treated genomic DNA, and water. PCR cycling conditions were 95°C for 60s, 57°C for 60 s and 72°C for 60 s for 50 cycles. PCR products were purified and sequenced by pyrosequencing as previously described [54] using 0.3 µΜ sequencing primer. Primers for *Alu*, *LINE-1, p16* and *p53* assay are shown in Table 4.

**Table 4:** Primers used for DNA methylation analysis.

ID	Forward Primer	Reverse Primer	Sequencing Primer	Sequence analyzed^a^
	*(5′ to 3′)*	*(5′ to 3′)*	*(5′ to 3′)*	
Global methylation analysis				
* Alu*	Biotin-TTTTTATTAAAAATATAAAAATT	CCCAAACTAAAATACAATAA	AATAACTAAAATTACAAAC	G/AC/TG/AC/TG/ACCACCA
* LINE–1*	TTTTGAGTTAGGTGTGGGATATA	Biotin-AAAATCAAAAAATTCCCTTTC	AGTTAGGTGTGGGATATAGT	TTC/TGTGGTGC/TGTC/TG
Gene-specific methylation analysis				
* p16*	AGGGGTTGGTTGGTTATTAG	Biotin - CTACCTACTCTCCCCCTCTC	GGTTGGTTATTAGAGGGT	GGGGC/TGGATC/TGC/TGTGC/TGTTC/TGGC/TGGTTGC/TG
* p53*	Biotin -TTAGGAGTTTATTTAATTTAGGGAAG	TATCCAACTTTATACCAAAAACCTC	TCCAAAAAACAAATAACTACTAAACTC	CG/AAAAACACTTTACG/ATTCG/AAACTAAAAACG/ATACTTT

a Nucleotides at which DNA methylation was measured are underlined.

In total, 120 paired maternal-umbilical cord blood samples underwent DNA methylation analysis. The %5mC was measured in *LINE-1, Alu*, seven specific positions in *p16*, and four specific positions in *p53*. The success of pyrosequencing ranged from 100% for *Alu* in maternal blood to 79% for *p53* in umbilical cord blood. For those assays that were unsuccessful, the paired maternal-umbilical cord sample was excluded from analysis.

### Statistical Analysis

Descriptive statistics were calculated for the maternal and cord blood samples. Differences between umbilical cord blood and maternal blood %5mC were evaluated using Wilcoxon-Rank Sum Tests. Spearman correlations coefficients that adjusted for drinking water arsenic exposure (and between batches using a dummy variable for *LINE-1* and *Alu*) were calculated to evaluate the association between %5mC in umbilical cord blood and maternal blood. Multiple linear regression models were used to evaluate the relationship between %5mC in umbilical cord blood (dependent variable) and maternal blood (predictor) for each marker. All regression models included drinking water arsenic exposure, infant sex and maternal age. The residuals from all regression models were evaluated for normalcy. Bonferroni correction was used to set the type I error rate at α = 0.05/7 = 0.007 for p16 and α = 0.05/4 = 0.01 for p53. This is a conservative approach that should reduce the potential for false positives associated with quantifying CpG methylation at 7 positions within *p16* and 4 positions within *p53*. All analyses were performed using SAS version 9.1 (SAS Institute Inc., Cary, NC, USA).
